# P02.172. 1-year sustaining efficacy of multidimensional therapy for inpatients with different conditions of chronic musculoskeletal pain

**DOI:** 10.1186/1472-6882-12-S1-P228

**Published:** 2012-06-12

**Authors:** R Stange, U Hackermeier, G Franzen, T Ostermann, B Uehleke, A Michalsen

**Affiliations:** 1 Charité, University of Medicine and Immanuel Hospital, Berlin, Germany; 2 Center of Integrative Medicine, Dprtmt. of Medicine, Witten, Germany

## Purpose

Chronic pain is the predominant condition for use of complementary medicine in Western countries. While many modalities have proven efficacy as a single intervention, therapeutical failure is often due to longer histories, comorbidities or complex etiologic conditions. Therefore, individually tailored therapies may show more efficacy, but little research could be retrieved. We investigated outcomes of inpatients after an intensive period of approximatey 2 weeks with individual combinations of therapies, composed of diet and fasting, physical therapy, relaxation, herbs, acupuncture, and neural therapy.

## Methods

Ongoing inpatients (age 18 – 70, M and F) with chronic musculoskeletal pain of different origin (Gerbershagen classification III and IV, multi-morbidity allowed) for more than 2 years were included. Main outcome parameters were changes in VAS (0-100) for global pain and SF-36 between T1 before therapy and final visit T5 after 1 year with no further protocol treatment. Results of this pivotal uncontrolled trial were interpreted as descriptive.

## Results

Two hundred twenty-one patients (intent to treat, mean age 57.2+12.2y) were enrolled with full data sets from 101 (per protocol). Most frequent diagnoses were low back pain (n=53, 24.0%), fibromyalgia (44, 19.9%), rheumatoid arthritis (25, 11.3%), and chronic neck pain (22, 10.0%). Mean VAS decreased by 15.1 from 60.7+23.0 (T1) to 45.6+26.2 (T5) (p<0.0001, two-sided t-test), with highest improvement for low back pain (decrease of 17.5) and no differentiation for multi-morbidity (n=46 with, n=55 without). SF-36 physical and mental component scores improved significantly from 40.0+12.2 to 44.3+12.5 and from 29.6+8.2 to 32.9+10.5, respectively (p<0.0001 for each).

## Conclusion

An individual multidimensional treatment for chronic musculoskeletal pain with inpatients may result in sustaining beneficial effects over one year. Surprisingly, differential improvement in quality of life extended to both physical and emotional dimensions. Non-compliance was attributed to the long interval between the end of therapy and visit for primary outcome measurement.

